# The ketogenic diet corrects metabolic hypogonadism and preserves pancreatic ß-cell function in overweight/obese men: a single-arm uncontrolled study

**DOI:** 10.1007/s12020-020-02518-8

**Published:** 2020-10-15

**Authors:** Sandro La Vignera, Rossella Cannarella, Fabio Galvano, Agata Grillo, Antonio Aversa, Laura Cimino, Cristina M. Magagnini, Laura M. Mongioì, Rosita A. Condorelli, Aldo E. Calogero

**Affiliations:** 1 Department of Clinical and Experimental Medicine, University of Catania, Catania, Italy; 2 Department of Biomedical and Biotechnological Sciences, University of Catania, Catania, Italy; 3 Labogen (Specialized Human Genetics Laboratory), Catania, Italy; 4 Department of Experimental and Clinical Medicine, University Magna Graecia of Catanzaro, Catanzaro, Italy

**Keywords:** VLCKD, Proinsulin, Insulin, β-cell dysfunction, Metabolic hypogonadism, Testosterone

## Abstract

**Background:**

Overweight and obesity are increasingly spread in our society. Low testosterone levels are often present in these patients, the so-called metabolic hypogonadism, that further alters the metabolic balance in a sort of vicious cycle. Very low-calorie ketogenic diet (VLCKD) has been reported to efficiently reduce body weight, glycaemia, and the serum levels of insulin, glycated hemoglobin, but its effects on β-cell function and total testosterone (TT) levels are less clear.

**Aim:**

To evaluate the effects of VLCKD on markers suggested to be predictive of β-cell dysfunction development, such as proinsulin or proinsulin/insulin ratio, and on TT values in a cohort of overweight or obese nondiabetic male patients with metabolic hypogonadism.

**Methods:**

Patients with overweight or obesity and metabolic hypogonadism underwent to VLCKD for 12 weeks. Anthropometric parameters, blood testing for the measurement of glycaemia, insulin, C-peptide, proinsulin, TT, calculation of body-mass index (BMI), and HOMA index were performed before VLCKD and after 12 weeks.

**Results:**

Twenty patients (mean age 49.3 ± 5.2 years) were enrolled. At enrollement all patients presented increased insulin, HOMA index, C-peptide, and proinsulin levels, whereas the proinsulin/insulin ratio was within the normal values. After VLCKD treatment, body weight and BMI significantly decreased, and 14.9 ± 3.9% loss of the initial body weight was achieved. Glycaemia, insulin, HOMA index, C-peptide, and proinsulin significantly decreased compared to pre-VLCKD levels. Serum glycaemia, insulin, C-peptide, and proinsulin levels returned within the normal range in all patients. No difference in the proinsulin/insulin ratio was observed after VLCKD treatment. A mean increase of 218.1 ± 53.9% in serum TT levels was achieved and none of the patients showed TT values falling in the hypogonadal range at the end of the VLCKD treatment.

**Conclusions:**

This is the first study that evaluated the effects of VLCKD on proinsulin, proinsulin/insulin ratio, and TT levels. VLCKD could be safely used to improve β-cell secretory function and insulin-sensitivity, and to rescue overweight and obese patients from β-cell failure and metabolic hypogonadism.

## Introduction

Overweight and obesity are characterized by an excess of fat-mass accumulation. They represent two widely spread conditions, whose prevalence doubled since the 1980s, currently affecting one-third of the global population [1]. There is a tight connection between obesity and the development of comorbidities such as type 2 diabetes mellitus (T2DM), dyslipidemia, cerebrovascular and cardiovascular disease, arthrosis, psychiatric disorders, obstructive sleep apnea, cholelithiasis, and non-alcoholic fatty liver disease. Thus, it heavily affects the quality of life and severely burdens the healthcare.

In the recent decade, male hypogonadism has been fully recognized as a complication of overweight and obesity, and the term “metabolic hypogonadism” has been coined. Particularly, the reduction of sex hormone-binding globulin (SHBG) serum levels, the increased androgen aromatization into estrogens, and the pro-inflammatory cytokine-dependent attenuation of luteinizing hormone (LH) pulses [2] represent some of the mechanisms explaining the occurrence of male hypogonadism in overweight and obesity [3]. Vice versa, low testosterone levels can promote adipocyte proliferation [4] and influence the pancreatic β-cell function [5]. Consequently, hypogonadism can worsen obesity and favor the development of metabolic diseases in overweight and obese patients, creating a sort of vicious circle.

Several therapeutic approaches have been developed to face up overweight and obesity, such as diet or weight-loss medications, although with documented poor efficacy, side effects, and restricted indications [6]. Bariatric surgery seems to have positive results in severely obese patients with or without comorbidity [7] and appears to reverse both hypogonadism [8] and metabolic abnormalities [9].

Ketogenic diets (KDs) are high-fat, normal-protein, and low-carbohydrate diet protocols firstly introduced in the 1920s for the treatment of refractory epilepsy, due to the benefits of ketone bodies (D-3-β-hydroxybutyrate, acetoacetate, and acetone) on epileptic seizures [10]. The principle of KD is a marked restriction of carbohydrate intake that pushes the energy supply from the β-oxidation of fatty acids, thus synthesizing acetyl-CoA. In the case of low glucose availability, acetyl-CoA escapes from the tricarboxylic acid cycle and contribute, by condensation, to the formation of ketone bodies [11]. The marked lipolysis that occurs in KD led to the idea that these protocols could be used for fat loss. Therefore, the “Atkins” diet and other similar low-calorie and low-carbohydrate KDs were subsequently developed with enough protein content for the maintenance of lean body mass and thus obtain rapid weight loss with lean mass savings [12–16]. These programs, which form the basis of VLCKD, are safe and have proven to have beneficial effects on blood pressure, glycaemia, and lipid profile [17]. Currently, VLCKD is defined by a document of the Italian Society of Endocrinology as a dietary protocol characterized by a marked restriction of daily carbohydrate intake (<30 g/day, approximately corresponding to ~13% of the daily energy intake), a 1.2–1.5 g/Kg protein intake (~43%) (which is not a high-protein content) and vegetable-derived (mainly unsaturated) fat intake (~44%), providing an overall total energy intake lower than 800 kcal [11].

Although VLCKD has been reported to efficiently reduce body weight, the levels of glycaemia, insulin, and glycated hemoglobin (HbA1c) in patients with overweight or obesity [18, 19], its effects on β-cell function are less clear. Also, despite VLCKD has been suggested for the treatment of metabolic hypogonadism [11], according to the authors’ knowledge, no study documented the effect of this diet protocol on total testosterone (TT) serum levels, so far. Therefore, this study aimed to evaluate the effects of VLCKD on β-cell markers suggested as predictive of β-cell dysfunction development (e.g., proinsulin or proinsulin/insulin ratio [20–23]) and on TT values in a cohort of overweight or obese nondiabetic male patients with metabolic hypogonadism.

## Subject and methods

### Ethical aspects

The experimental protocol was designed to be an observational prospective study. It was performed in the Division of Andrology and Endocrinology of the Teaching hospital “G. Rodolico”, University of Catania, Catania, Italy. The internal Institutional Review Board approved the study protocol. An exhaustive explanation of the study purpose was given to each participant and informed written consent was obtained in compliance with Helsinki’s declaration.

### Patient selection

Male patients with overweight or obesity, older than 18 years, consulting our Division for weight loss, underwent comprehensive medical history collection, physical examination, and laboratory testing. Patients with signs or symptoms suggestive of T deficiency underwent to TT measurement. Those with hypogonadism (TT < 264 ng/dl confirmed by at least two measurements of serum TT [22]) willing to undergo to VLCKD were recruited in this study.

The exclusion criteria were given by the presence of the following [8]: type 1 diabetes mellitus, latent autoimmune diabetes, T2DM, chronic renal failure with estimated glomerular filtration rate <60 ml/min/1.73 m^2^, active or severe infections, recent major cardiovascular event, unstable angina, cardiac arrhythmias, frailty, 48 h prior surgery or invasive procedures, eating disorders and other psychiatric disturbances. Also, patients with hypertension, dyslipidemia, and users of drugs for the treatment of chronic diseases in the 3 months before enrollment were excluded. Finally, patients with hypogonadism due pituitary or testicular diseases were excluded.

### Diet protocol

VLCKD protocol was based on marked restriction of daily carbohydrate intake (<30 g/day), a fat intake of ~44%, and ~43% was the intake of proteins. Particularly, 1.4–1.5 g/Kg/daily protein of the ideal body weight was chosen for the patients enrolled in this study. The protocol consisted of five main phases. The first (VLCKD: 600–800 kcal/day) was based on a total replacement of natural proteins with five substitutive meals (breakfast, lunch, dinner, and two snacks, one mid-morning and one mid-afternoon). Vegetables with low glycemic index were allowed during lunch and dinner. Two different programs could be chosen in the second phase (low-calorie ketogenic diet: 800–1000 kcal/day). The first was replacement of a single protein preparation with a natural protein food (lunch or dinner), such as meat, eggs, and fish; the second option was keeping breakfast and snacks with protein preparations and replace both lunch and dinner with natural proteins. Only vegetables with a low glycemic index were allowed in both phases. These two phases had a 12-week-long duration during which ketosis was maintained. Micronutrients, consisting of vitamins (complex B, C, and E), minerals (sodium, potassium, magnesium, and calcium) and omega-3 fatty acids, were supplemented. The third phase (low-calorie diet: 1200–1500 kcal/day) was characterized by gradual carbohydrate replenishment, based on their glycemic index. Protein preparations were also progressively replaced with natural foods and vegetables with a higher glycemic index were reintroduced. Protein preparations were used for breakfast and one snack, while the other snack was replaced by fruit. Meat, eggs, fish, and dairy products were introduced 3–4 times a week for lunch and dinner. In this phase, at least 10 min of physical activity per day were suggested. Pasta or bread (lunch) and cereals (breakfast or dinner) and, finally, legumes (lunch or dinner) were reinstated in the fourth and fifth phases (total daily calorie intake between 1500 and 2000 kcal/day, depending on the individual). 150 min/week of physical activity was recommended in these phases to achieve body weight control. In the present study, each patient underwent to the diet protocol for at least 12 weeks (which included the 1st and the 2nd phases). All patients voluntarily referred to Therascience Lignaform (Monaco, France), for the purchase of substitutive meals.

### Anthropometric parameters and hormonal measurements

Anthropometric and biochemical parameters were evaluated at enrollment and at the end of the second phase (after 12 weeks from the beginning of VLCKD). Beta-hydroxybutyrate levels were detected weekly through a reflex metrics detection system and kept between 0.5 and 0.7 mmol/l.

Body weight was measured in fasting conditions, using the same calibrated scale, without shoes, and with an empty bladder. Each man underwent to blood testing for the measurement of glycaemia, insulin, C-peptide, proinsulin, and TT. The blood samples were collected in the morning (8–10 a.m.), in a fasting state. Laboratory evaluation was performed by electrochemioluminescence (ECLIA) (Roche Cobas, Germany) for plasma glucose, insulin C-peptide and TT. Serum proinsulin was determined by immunosorbent assay (Mercovia, Biomerieux, France).

The reference values were as follows: glycaemia 60–100 mg/dl [intra-assay coefficient of variation (CV) 1.7%; inter-assay CV: 1.2%], insulin <25 µIU/ml (intra-assay CV 1.7%; inter-assay CV: 1.2%), C-peptide 0.8–4 ng/ml (intra-assay CV 2.1%; inter-assay CV: 1.6%), proinsulin <11 pmol/l (intra-assay CV: 4.8%; inter-assay CV: 5.9%), TT 300–1000 ng/dL (intra-assay CV 4.6%; inter-assay CV: 2.1%). The homeostasis model assessment (HOMA) index was evaluated using the formula: [glycaemia (mg/dl) × insulin (µIU/ml)]/405, and those values falling into the 0.23–2.5 range were considered as normal. The proinsulin/insulin ratio was calculated using the formula: proinsulin (pmol/l)/[insulin (µIU/ml) × 7.175], where 7.175 is the conversion factor for µIU/ml to pmol/l, as shown elsewhere [24].

### Statistical analysis

Results are reported as mean ± SD for continuous variables throughout the study. Normality was evaluated using the Shapiro–Wilk test and all variables resulted normally distributed. Analysis of data was hence performed by the Student *t* test. Statistical analysis was performed using SPSS 22.0 for Windows (SPSS Inc., Chicago, IL, USA). A *p* value of < 0.05 was accepted as statistically significant. A trend was assumed for *p* values ranging from 0.05 to 0.099.

## Results

Applying the above-mentioned exclusion criteria, 38 patients were initially considered for inclusion. Eighteen patients were excluded for low compliance to the VLCKD protocol (*n* = 8) and recurrent fever (*n* = 2). Eight patients quitted for economic reasons. Therefore, 20 patients (mean age 49.3 ± 5.2 years) were ultimately enrolled in this study. All patients completed the VLCKD protocol and no drops out were registered. At enrollment, the mean weight and BMI were 93.0 ± 6.5 Kg and 32.0 ± 3.1 Kg/m2, respectively. Proinsulin/insulin ratio (0.04 ± 0.01) was lower than the values suggested to predict ß-cell exhaustion [21, 22]. Also, the mean fasting glycaemia (93.6 ± 13.5 mg/dl) was within the normal range, and 40% of patients had impaired fasting glucose (≥100 mg/dl). By contrast, the entire cohort showed elevated insulin levels (40.4 ± 7.1 µIU/ml), HOMA index (9.3 ± 2.2), and C-peptide levels (5.2 ± 1.1 ng/ml), indicating the presence of insulin-resistance, and elevated serum proinsulin levels (12.6 ± 1.1 pmol/l), suggesting β-cell dysfunction. Serum TT levels (177.7 ± 36.5 ng/dl) showed the presence of hypogonadism in all patients, as for the inclusion criterion. Mean serum LH levels were 2.6 ± 0.6 IU/l.

**Fig. 1:**
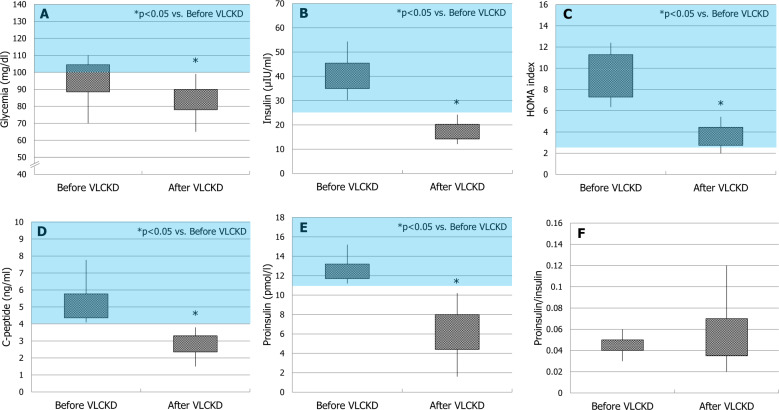
Metabolic parameters before and after very low-calorie ketogenic diet (VLCKD). The pink rectangles indicate the range of values considered abnormal (≥100 mg/dl for glycaemia, ≥25 µIU/ml for insulin, ≥2.5 for HOMA index, ≥4 ng/ml for C-peptide, and ≥11 pmol/l for proinsulin). At enrollment, values of insulin (**b**), HOMA index (**c**), C-peptide (**d**), and proinsulin (**e**) were outside the normal range in all patients. With the exception for proinsulin/insulin ratio (**f**), all values decreased significantly after VLCKD compared to their respective values at enrollment (**a**–**e**). The end of diet glycaemia (**a**), insulin (**b**), C-peptide (**d**), and proinsulin (**e**) levels returned within the normal range in all patients

**Fig. 2:**
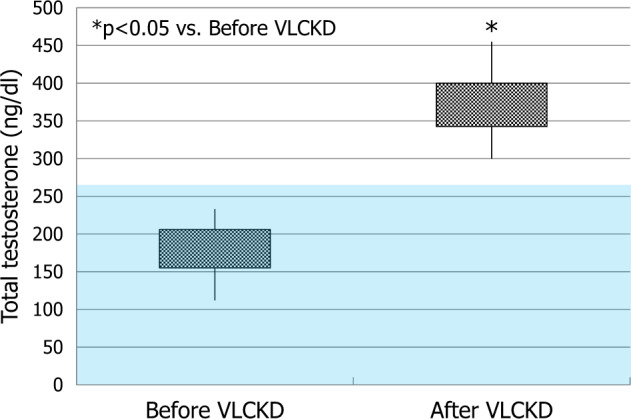
Baseline and end of diet total testosterone (TT) levels. TT values increased significantly after very low-calorie ketogenic diet (VLCKD). The pink rectangle indicates values lower than 264 ng/dl, cutoff for the diagnosis of hypogonadism according to the last Endocrine Society guidelines (Bhasin et al., 2018). At enrollment, all patients had metabolic hypogonadism. None of the patients showed end of diet TT values falling within the hypogonadal range

A significant decrease of body weight (79.1 ± 5.8 Kg) and BMI (27.2 ± 2.8 Kg/m2) (*p* < 0.01) was observed after VLCKD treatment. The patients overall lost 14.9 ± 3.9% of the initial body weight. Glycaemia (83.5 ± 10.6 mg/dl), insulin (17.6 ± 3.8 µIU/ml), HOMA index (3.7 ± 1.0), C-peptide (2.8 ± 0.7 ng/ml), and proinsulin (6.2 ± 2.5 pmol/l) significantly decreased (*p* ≤ 0.01) compared to pre-VLCKD levels. Notably, serum glycaemia, insulin, C-peptide, and proinsulin levels returned within the normal range in all patients. No difference in proinsulin/insulin ratio was observed (0.05 ± 0.03, *p* > 0.1) after VLCKD (Fig. 1). TT significantly increased (371.2 ± 43.7 ng/dl, *p* < 0.01) and none of the patients showed TT values falling in the hypogonadal range following VLCKD; Overall, TT levels increased by the 218.1 ± 53.9%. Thus, VLCKD rescued from metabolic hypogonadism (Fig. 2).

## Discussion

The present study was conducted to evaluate the effects of VLCKD on pancreatic ß-cell function and TT levels in a cohort of overweight/obese nondiabetic male patients with metabolic hypogonadism. The diagnosis of hypogonadism was made according to the latest guidelines of the Endocrine Society [25].

Overweight and obese subject lead the ß cells of the pancreas to a secretory effort, as they are called to release a greater amount of insulin to maintain glucose levels within the physiological range. Accordingly, hyperinsulinemia is present in the vast majority of obese patients [26]. Overweight/obesity causes the development of insulin-resistance in peripheral tissues, which, in turn, increases even more the release of insulin. The secretory effort of the β-cells is responsible for the onset of a progressive functional failure that ultimately results in T2DM. Markers of β-cell dysfunction have been proposed as more reliable predictors of T2DM development in the Caucasian population than markers of insulin-resistance [27].

Proinsulin, an insulin precursor with glucose-lowering effects, is one of the proposed markers. Ancient double-blind phase II studies aimed at assessing the possible therapeutic role of proinsulin were prematurely interrupted due to the occurrence of macrovascular events in the proinsulin arm, in newly diagnosed patients with DM [28]. This supported the contention that proinsulin has adverse effects. Indeed, similarly to insulin, it can trigger the MAP-kinase pathway, leading to macrovascular disease [28]. Thus, the positive correlation between proinsulin and premature coronary artery disease has already been established [29–31]. Proinsulin is released by pancreatic β-cells in an increasing manner when they reach a late stage of deterioration [32, 33], as a further glucose-lowering tentative to delay the onset of T2DM. Large population studies with a duration of up to 27 years reported increased serum proinsulin levels as predictors of T2DM development within 2–7 years [27, 34–36]. More recently, the cutoff value of proinsulin ≥7.829 pmol/l has been reported to be a predictor of β-cell dysfunction with a sensitivity of 95.8% and a specificity of 72.2% in a cohort of obese adolescents [22].

A disproportionate secretion of proinsulin has been described in patients with T2DM2 and it has been implied as a strong predictor of β-cell dysfunction and insulin-resistance [37]. Some authors have attempted to find a cutoff value of the proinsulin/insulin ratio. In a case-control study on 50 patients with T2DM and diabetic nephropathy, the value of 0.1145 has been suggested to predict insulin-resistance with relatively high sensitivity (92.3%) but with low specificity (60.2%) [23]. Similarly, the value of 0.1545 has been reported as predictive of insulin-resistance with lower sensitivity (87.5%) but similar specificity (61.1%) in adolescents with obesity [22].

No study has investigated the effects of VLCKD on proinsulin and proinsulin/insulin ratio so far. All the patients enrolled in the present study showed high proinsulin levels, which could reveal the presence of β-cell dysfunction and a predisposition to T2DM development later on. After the VLCKD treatment, proinsulin mean value was lower than 7.829 pmol/l suggesting a rescue of β-cell dysfunction [22], and this also decreased the risk for the patients to develop proinsulin-induced macrovascular complications [28]. Pre-VLCKD proinsulin/insulin ratio in all patients felt within the normal range according to the available literature [22, 23]. This may explain why the proinsulin/insulin ratio did not change significantly at the end of the diet.

The effects of VLCKD on body-weight, glycaemia, insulin, and C-peptide, have been more widely investigated. A meta-analysis carried out on 1577 patients with obesity randomized in the low-fat diet (*n* = 787) and VLCKD (*n* = 790) (females were the majority in almost all the studies included in the meta-analysis) has shown significant decrease in body-weight and blood-pressure in subjects assigned to VLCKD than low-fat diet, but no significant difference was found for glucose, insulin, and HbA1c levels [18]. However, a subsequent meta-analysis on 801 adult patients with overweight or obesity treated with VLCKD and kept in ketosis for at least 4 weeks showed a significant amelioration of body-weight, lipid profile, HbA1c, blood pressure, and liver function [19]. The lowering effects of VLCKD on blood glucose, insulin, and HbA1c have been confirmed by several studies [38–41]. Benefits on insulin-resistance (measured by the HOMA index) and insulin-sensitivity (measured by HOMA β or the WBISI index) have also been reported [42–44] and are consistent with the results of the present study. In fact, we even found a normalization of glycaemia, insulin, C-peptide, and a significant decrease of the HOMA index, which is in line with previous studies that reported T2DM remission [38–41].

Our study was carried out in patients with hypogonadism. Low testosterone levels worsen peripheral insulin-resistance and therefore push toward the development of T2DM [45]. More in detail, testosterone can influence carbohydrate, protein, and lipid metabolism. Based on the hypogonadal–obesity–adipocytokine hypothesis, central obesity associates with lower testosterone levels due to the aromatization into estrogen occurring in adipocytes. In turn, the activity of lipoprotein lipase, involved in the storage of triglycerides into the adipocytes, and the differentiation of pluripotent stem cells into mature adipocytes are favored by the low testosterone levels [46]. The subsequent adipocyte accumulation and enlargement leads to insulin-resistance. Furthermore, pro-inflammatory cytokines released by adipocytes (e.g., TNFα, IL6) and the increase of circulating leptin and estrogens are all involved in the inhibition of kisspeptin neurons. This results in a decreased GnRH secretion and lower LH pulses and, therefore, in low LH-stimulated testosterone secretion. Also, leptin directly inhibits testosterone release from the Leydig cells [46]. These mechanisms underlie the pathogenesis of metabolic hypogonadism, occurring in up to half of the obese patients [47]. Thus, hypogonadism represents an additional risk factor in overweight and obese patients for the development of metabolic and cardiovascular complications [48]. Hence, restoration of testosterone levels is of great importance in patients with overweight or obesity to decrease the risk of developing long-term sequels.

A randomized controlled trial assessed the effects of a 10-week-long very-low-energy diet (VLED) protocol (without ketosis) on testosterone levels, describing a 30% increase compared to the values before diet [49]. This finding has also been confirmed elsewhere [50]. Another randomized placebo-controlled trial investigated the role of testosterone replacement therapy on body composition in 100 obese male patients on VLED. The authors concluded that the testosterone plus VLED arm of the study was associated with fat-mass loss, while the placebo plus VLED arm showed both fat- and lean-mass losses [51]. This highlights the importance of normal testosterone levels in the effects of diet on body composition. In the present study, we report the effects of VLCKD on TT levels. Metabolic hypogonadism is a functional and potentially reversible condition, which can benefit from diet, bariatric surgery, and even drugs such as selective estrogen receptor modulators (SERM) [8, 52–54]. At the best of our knowledge, this is the first study showing the effects of VLCKD on metabolic hypogonadism. Patients on VLCKD obtained a ~200% increase of serum TT levels and a complete remission from metabolic hypogonadism since none of the enrolled patients showed TT levels falling within the hypogonadal range after VLCKD. However, these results warrant confirmation in controlled double-arm studies to evaluate the effectiveness of VLCKD compared to VLCD.

Our results need to be taken with care. The small sample size and the absence of a control group represents limits of the present study and further double-blinded randomized controlled trials are warranted to confirm our findings. In addition, in the present study hypogonadism was defined for TT < 264 ng/dl, as the Endocrine Society guidelines recommend [25]. Neither measurement of SHBG and albumin, nor free-T calculation was available in patients with TT in “grey zone“ (264–300 ng/dl), which may have possibly impacted on the real prevalence of hypogonadism in the enrolled patients.

In conclusion, the present study explored the effects of VLCKD on body weight, indices of β-cell dysfunction, insulin-resistance, and on serum TT levels in overweight or obese male patients with metabolic hypogonadism. According to the available literature, we found that VLCKD was effective on body-weight and insulin-resistance. This was the first study to report the normalization of proinsulin and TT levels after 12 weeks of VLCKD-induced ketosis. This suggests the safety of ketonemia for β-cells and the effectiveness of VLCKD in restoring β-cell dysfunction and the gonadal function. Thus, VLCKD could be used to improve β-cell secretory function and insulin-sensitivity, and to rescue β-cells from exhaustion and metabolic hypogonadism.
